# Peripheral giant cell granuloma associated with a dental implant

**DOI:** 10.1186/s12903-019-0983-2

**Published:** 2019-12-16

**Authors:** Rafaela Carriço Porto Baesso, Maria Carolina de Lima Jacy Monteiro Barki, Rebeca de Souza Azevedo, Karla Bianca Fernandes da Costa Fontes, Débora Lima Pereira, Renata Tucci, Fábio Ramôa Pires, Bruna Lavinas Sayed Picciani

**Affiliations:** 1 Graduate Program in Dentistry, Health Institute of Nova Friburgo, Universidade Federal Fluminense, Nova Friburgo, Rio de Janeiro, Brazil; 2 Department of Specific Formation, School of Dentistry, Universidade Federal Fluminense, Nova Friburgo, Rio de Janeiro, Brazil; 3 Department of Oral Pathology, School of Dentistry, Universidade Estadual do Rio de Janeiro, Rio de Janeiro, Brazil; 4 Graduate Program in Pathology, Medical School, Universidade Federal Fluminense, Niterói, Rio de Janeiro, Brazil

**Keywords:** Peripheral giant cell granuloma, Dental implants, Oral lesion, Peri-implant lesions

## Abstract

**Background:**

Peripheral giant cell granuloma (PGCG) is an uncommon pathology that affects gingival or alveolar mucosa. Although PGCG can be associated with dental implants, little is known about this lesion and implant osseointegration as well as its etiopathogenesis and the treatments available. This study sought to report a rare case of PGCG associated with dental implant, emphasizing its clinical and histopathological aspects.

**Case presentation:**

A 53-year-old man had an exophytic, reddish lesion, around a crown attached to a dental implant located in the left mandible. Radiographically, there was bone loss around the implant. After excisional biopsy, histological examination revealed a submucosal proliferation of multinucleated giant cells rendering the diagnosis of peripheral giant cell granuloma. Patient has been under follow-up for 6 months with no recurrence.

**Conclusions:**

Peri-implant lesions must be completely removed to prevent recurrence of PGCG and implant failure, even in cases suspected to be reactive. Besides, histological examination must be performed on all peri-implant reactions to achieve the appropriate diagnosis and, consequently, the best treatment and follow up.

## Background

Peripheral giant cell granuloma (PGCG) is an uncommon pathology, which affects gingival or alveolar mucosa. Although the uncertain etiology, this lesion has been described as a reaction to chronic local injuries such as sub- or supragingival dental biofilm, ill-fitting restorations and dentures, and trauma [1]. It can occur either in dentate or edentulous area and the five main hypotheses suggest its origin can be from the periodontal ligament, the periosteum or the persistence of cells from periodontal ligament after tooth extraction. More frequently seen in individuals aged between the 3rd and 5th decades of life, it has a slightly higher prevalence in female subjects. Clinically, PGCG is a reddish or red-blue nodule with a fibrous consistency and sometimes an ulcerated surface. Most PGCG grow progressively and might result in dental displacement and resorption of the underlying alveolar bone [1, 2].

Currently, the presence of PGCG can be seen associated with dental implants, mostly due to unfitting angulation, gaps between the prosthetic crown and the screw, and peri-implantitis. Regarding diagnosis, treatment and prognosis, studies relating this lesion to implant osseointegration are scarce in the literature. Thus, publishing these cases is essential to better understand the PGCG etiopathogenesis when associated with dental implants and to avoid further damages such as implant loss. Our study sought to report a rare case of PGCG associated with a dental implant, pointing out its clinical and histopathological aspects [3].

## Case presentation

**Fig. 1:**
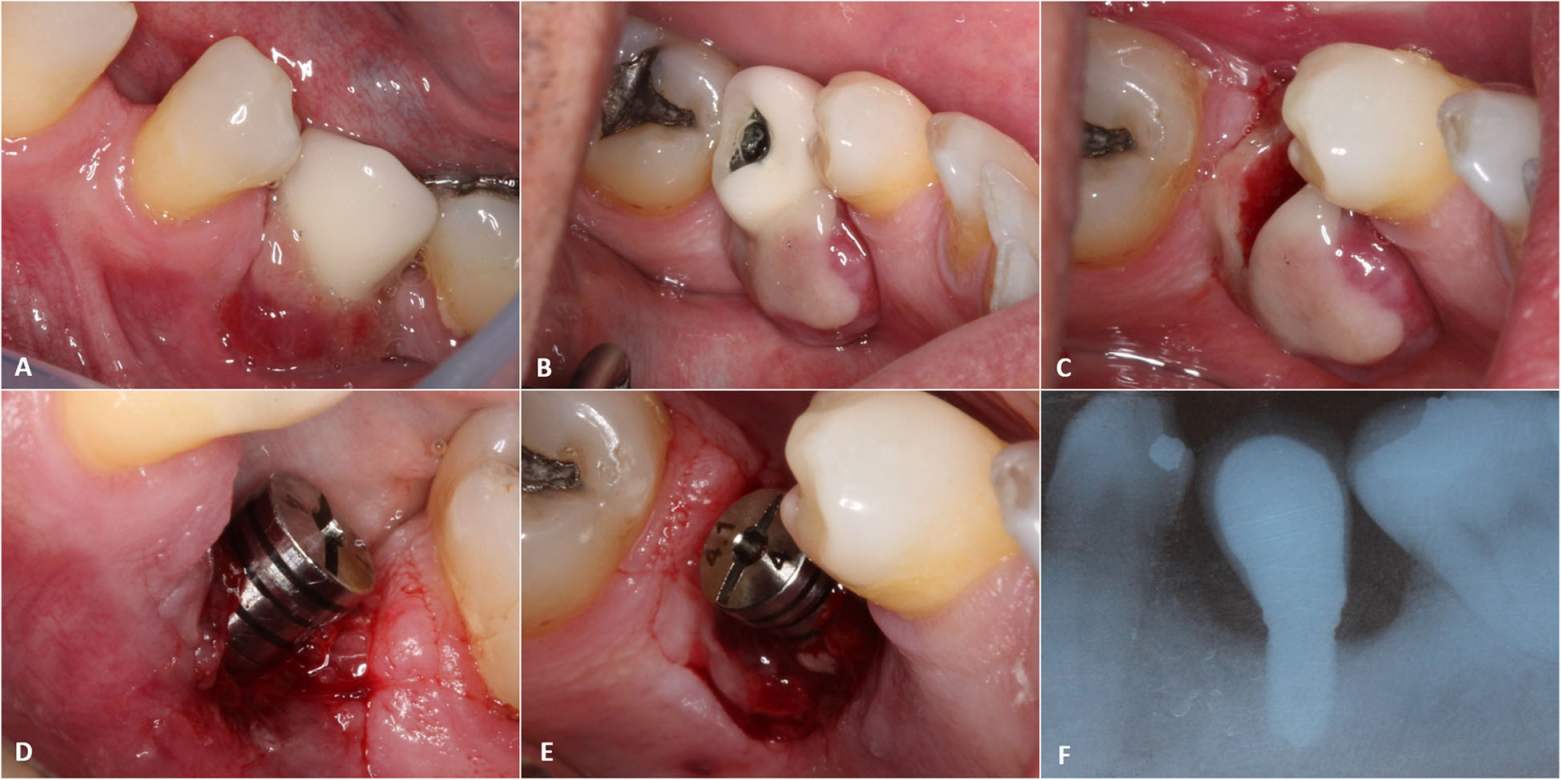
**a.** and **b**. Early clinical aspects: Reddish exophytic lesion around the prosthetic crown attached to dental implant. Buccal and lingual view. **c.** Clinical aspect after prosthetic crown removal. **d.** and **e.** The area after the excisional biopsy and placement of an abutment cover. **f.** Radiographic aspect after removing the lesion, showing good osseointegration of the dental implant, despite the bone loss

A 53-year-old Caucasian man was referred to the Service of Stomatology with a chief complaint of a lesion involving a dental implant. His medical history revealed controlled hypertension, using antihypertensive medication (Atenolol 50 mg). Intraoral exam showed an exophytic, pedunculated and reddish lesion with the lingual surface covered by a pseudomembrane, measuring nearly 5 × 1 cm (Fig. 1 a and b). The lesion had smooth surface, flaccid and friable consistency, bleeding on touch, involving the vestibular and lingual surface of the crown attached to the implant, which was placed in the region of the left inferior second premolar (Fig. 1 c). Radiographically, the patient presented a reduction of the whole bone level around the associated dental implant.

The hypotheses of peri-implant infection and pyogenic granuloma were diagnosed, and an excisional biopsy under local anesthesia was performed in association with curettage of the remaining surgical site. Moreover, the prosthetic crown was removed and replaced by a 4.1 × 4 abutment cover, and the patient was recommended to take anti-inflammatory for 5 days combined with application of 2% Chlorhexidine gel for 7 days (Fig. 1 d and e).

**Fig. 2:**
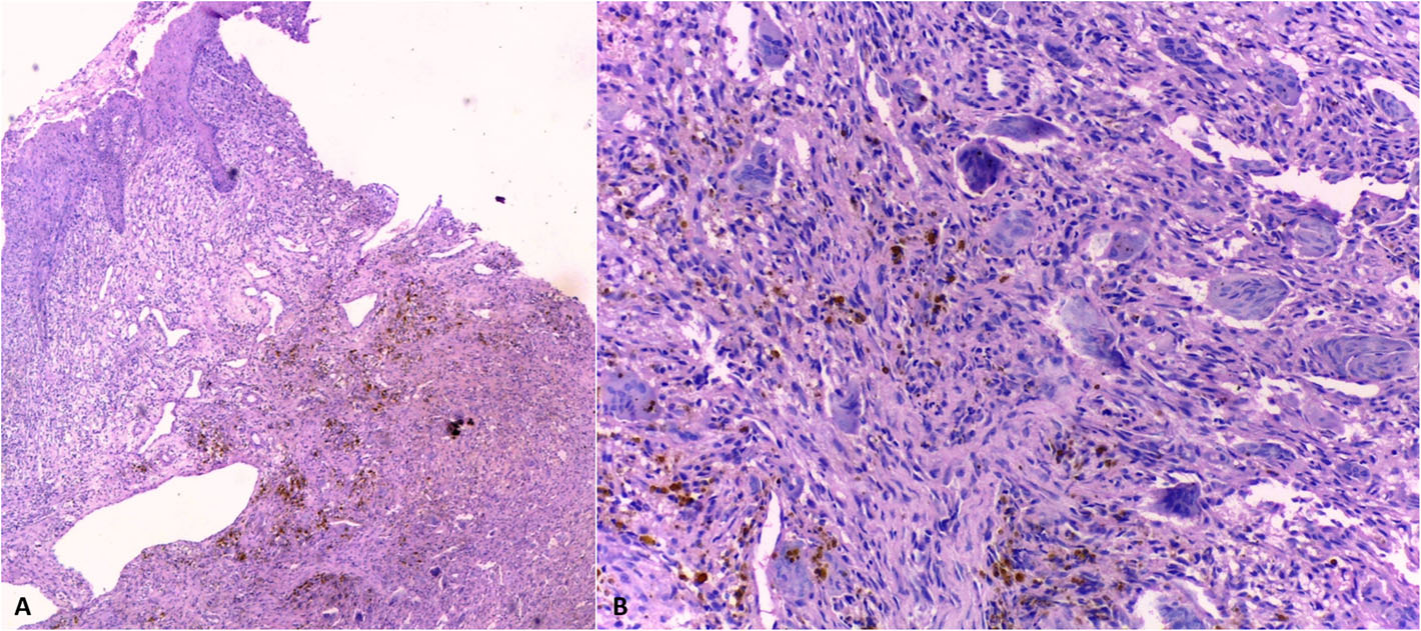
Histopathological features: ***a.*** *mucosa* showing non-keratinized and hyperplastic stratified squamous epithelium non-keratinized and hyperplastic cells with moderate inflammatory infiltrate and vascularization in the underlying lamina propria surface. **b.** Higher magnification of a cellular area emphasizing the presence of multinucleated giant cells permeated by mononuclear inflammatory cells in fusiform and ovoid shape. Note the presence of hemosiderin deposits near the area (HE, 200x)

Histological exam showed mucosa surface composed of a non-keratinized and hyperplastic stratified squamous epithelium, exhibiting intense inflammatory infiltrate and vascularization in the underlying lamina propria. Proliferation of multinucleated giant cells permeated by mononuclear inflammatory cells in fusiform and ovoid shape was observed. In the depth, we could observe hemosiderin deposition and a thick cellular connective tissue (Fig. 2 a and b). The histological exam was compatible with the diagnosis of PGCG.

Additionally, 2 sessions of photodynamic therapy (methylene blue solution 0.01%, 4 J and a low-level laser 660 nm), including an interval of one week between them, was done to remove the bacterial colonization and biofilm control. Finally, the prosthetic crown was replaced.

**Fig. 3:**
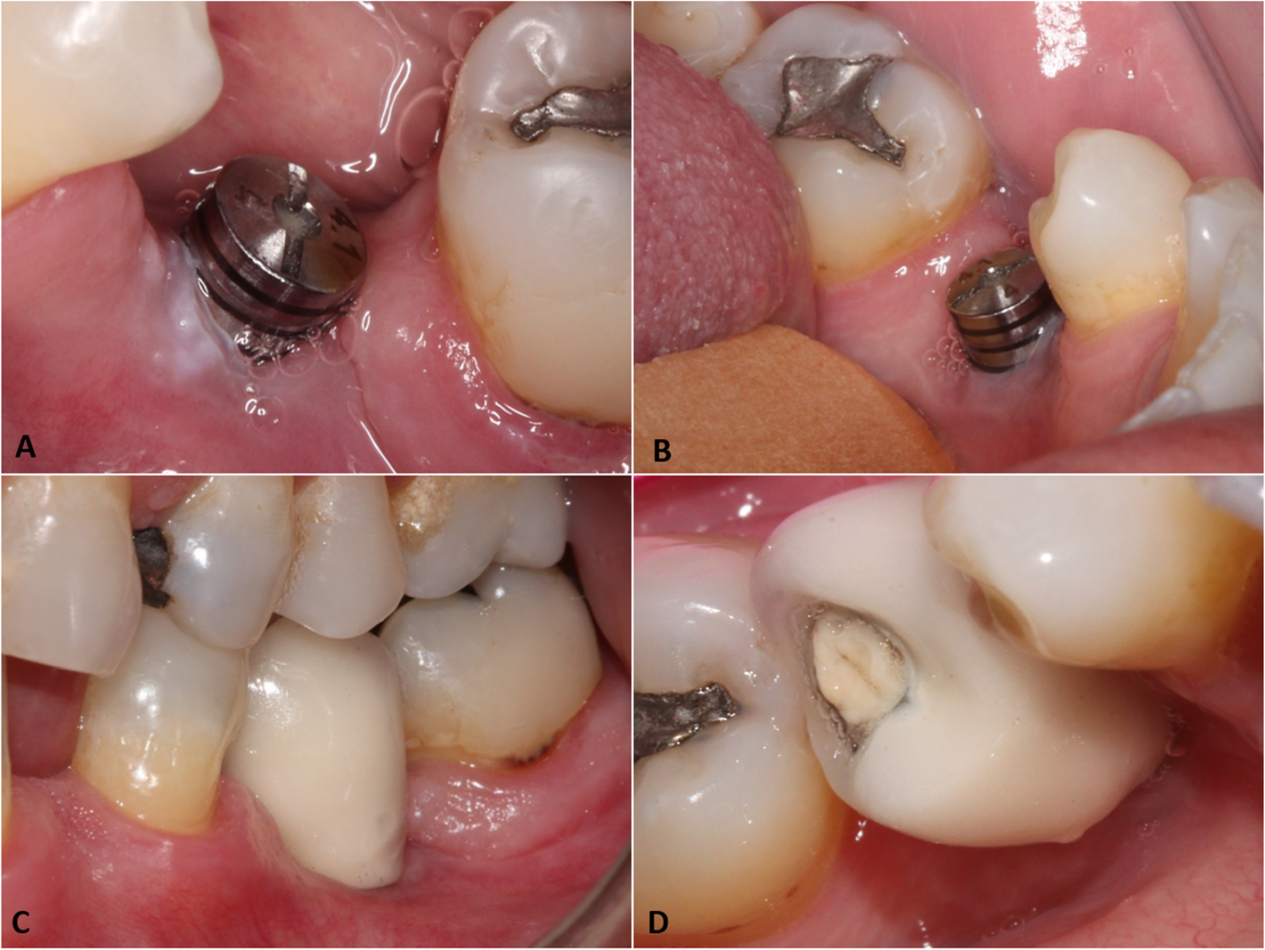
Follow-up after lesion removal: **a.** and **b.** vestibular and lingual views of the lesion after 30 days. **c.** and **d.** vestibular and lingual views of the lesion 90 days after surgery and placement of prosthetic crown

There were no complications during the postoperative period (Fig. 3 a and b) and the patient has been under follow-up for 6 months without signs of recurrence (Fig. 3 c and d). The study was approved by the Research Ethics Committee of the School of Medicine, Universidade Federal Fluminense (95,988,618.9.1001.5243). The patient signed an Inform Consent Form.

## Discussion and conclusions

PGCG and pyogenic granuloma (PG) are reactive hyperplastic lesions associated with natural teeth or implants. Dental implants can develop complications such as gingival hyperplasia, mucositis and, sometimes, peri-implantitis [1, 3, 4]. In natural teeth, a recent study on the prevalence of PGCG and PG revealed that 49% of all specimens adjacent to teeth received by pathology laboratories consist in one of these two lesions. The first group to report cases of PGCG involving dental implants was Hirshberg et al. in 2003, with 2 cases in the posterior mandible and 1 in the anterior maxilla [2, 5].

Only 19 cases of PGCG around osseointegrated implants, including the case we studied, are reported in the literature in English (Table 1). The exact etiology is still unknown, although the literature consensually establishes that the lesion peri-implant PGCG is originated from the periosteum or the periodontal ligament after a chronic local irritation and that the giant cells derive from osteoclasts [1, 3]. Moreover, is has been shown that poor implant angulation, narrow band of keratinized mucosa, inefficient abutment covers, poor local hygiene and ill-fitting prosthetics influence the development of these lesions, especially PGCG in areas with unsuitable dental implants [4, 14]. In the case we studied, there was a gap between the two components, compromising a proper hygiene, regardless of the good condition of the prosthetic crown attached to the implant.

**Table 1:** Clinical features of dental implant-associated PGCG cases reported in literature

Author (year)	Sex	Age (years)	N implants	Localization	Bone loss	Implant preservation	Treatment	Recurrence	Follow-up
Hirshberg et al. (2003) [2]	Male	31	–	Posterior mandible	Yes	Yes	Lesion removal	Yes	–
Hirshberg et al. (2003 )[2]	Female	69	–	Anterior maxilla	No	No	Lesion + Implant removal	Yes	–
Hirshberg et al. (2003 )[2]	Male	44	–	Posterior mandible	Yes	No	Lesion + Implant removal	Yes	–
Bischof et al. (2004 )[4]	Female	56	1	Posterior mandible	No	Yes	Lesion removal + new prosthetic + Biofilm control	No	36 months
Cloutier et al. (2004)[6]	Male	21	1	Posterior mandible	Yes	No	Lesion + Implant removal	No	12 months
Scarano et al. (2008)[7]	Female	48	1	Posterior maxilla	Yes	Yes	Lesion removal + tissue graft	No	–
Hernández et al. (2009)[8]	Female	62	3	Posterior mandible	Yes	Yes	Lesion removal	No	2 months
Hernández et al. (2009)[8]	Female	45	3	Posterior mandible	Yes	1 implant lost	Lesion removal + curettage	No	108 months
Hernández et al. (2009)[8]	Female	36	1	Posterior maxilla	Yes	No	Lesion + Implant removal	Yes	12 months
Ozden et al. (2009 )[7]	Female	60	2	Posterior mandible	Yes	Yes	Lesion removal + new prosthetic	No	12 months
Olmedo et al. (2009)[9]	Female	64	2	Anterior maxilla	Yes	Yes	Lesion removal + curettage	No	24 months
Peñarrocha-Diago (2012 )[10]	Female	54	1	Posterior mandible	Yes	Yes	Lesion removal + curettage + scale of implant	No	12 months
Pacific et al. (2015 )[11]	Male	60	1	Anterior maxilla	Yes	Yes	Lesion removal + curettage	No	19 months
Brown et al. (2015 )[3]	Male	46	1	Posterior mandible	No		Lesion removal + curettage + surgical cement	Yes	12 months
Galindo-Moreno et al. (2016 )[12]	Male	76	2	Posterior maxilla	Yes	Yes	Lesion removal	No	6 months
Scarano et al. (2017)[13]	Male	26	1	Posterior mandible	Yes	No	Lesion + Implant removal	No	12 months
Scarano et al. (2017)[13]	Female	52	1	Posterior maxilla	Yes	No	Lesion + Implant removal	No	12 months
Scarano et al. (2017)[13]	Male	31	3	Anterior mandible	Yes	No	Lesion + Implant removal	No	12 months
Present case	Male	56	1	Posterior mandible	Yes	Yes	Lesion removal + curettage	No	6 months

Contrastingly with our case, a slightly higher prevalence in females has been observed in the literature. Some authors suggest this higher prevalence might be explained by the hormonal influence over multinucleate giant cells, which are a target of estrogen [3, 4]. Regarding the patient’s age, PGCG often affects people between the 3rd to 5th decade of life, which agrees with our case.

The PGCG is invariably found in gingival or alveolar ridge mucosa [4] more often in the anterior mandible. Clinically, it presents as an exophytic lesion with a smooth surface, reddish-purple coloring, sessile or pedunculated basis, and firm-elastic consistency, frequently asymptomatic. Our case corroborates the characteristics described in the literature, except for being found in the posterior region, possibly due to the loss of posterior teeth being more common than of anterior teeth. This affirmative also justified the similar predominance of posterior region in PGCG associated with dental implants.

Periapical radiographic exam of a PGCG lesion affecting dentate areas may evidence resorption of the alveolar bone, widening of the periodontal ligament space and, rarely, root resorption. We could observe a bone resorption in a concave shape in the edentulous area. Figure 1f shows a bone resorption around the dental implant and the poor angulation of the prosthetic piece [15] as most cases associated with implants reported in the literature.

Histologically, PGCG is characterized by stratified squamous epithelium that may be atrophic or hyperplastic, containing moderate inflammatory infiltrate and vessels in the superficial lamina propria. In the connective tissue, it is evident a proliferation of multinucleated giant cells within a background of plump ovoid and spindle-shaped inflammatory cells, frequently with deposits of hemosiderin pigment throughout the tissue [10]. Figure 2a and b show these characteristics in combination with a hypercellular and dense fibrous connective tissue. Galindo-Moreno et al. affirm that there is a difference of immunophenotype among giant cells of PGCG, the giant cell reparative granuloma and the peri-implant osteolysis. Their findings suggest that giant cells share immunohistochemistry expression of monocytes/macrophages markers (CD68, acid phosphatase, cathepsin K, and microphthalmia associated transcription factor); however, this panel is not essential to determine diagnosis [12]. Besides the brown tumor, other reactive lesions can be clinically similar to PGCG such as PG and peripheral ossifying fibroma [1, 4].

Treatment of these lesions remains controversial in the literature. Some authors [2, 14–16] defend the removal of the dental implant for complete resolution of the lesion. On the other hand, other authors affirm that removing the biofilm, excising the lesion and curetting the region is usually sufficient for healing, and, sometimes, scaling the dental implants and replacing the prosthetic crown may also be necessary [3, 10, 12, 17]. The patient underwent a conservative treatment, including an excisional biopsy, curettage of the remaining surgical site, removal of the prosthetic crown and replacement by a 4.1 × 4 abutment cover. A careful curettage was performed, and the irritant factor was eliminated. Patient was indicated for confection of a new prosthetic crown. Additionally, 2 sessions of photodynamic therapy were done to remove the bacterial colonization and biofilm control. Patient remains in clinical and radiographic follow-up, presenting well recover with no evidences of recurrences of the PGCG.

Photodynamic therapy (PDT) has been used as a complementary treatment for peri-implant diseases. The strict etiologic relation between biofilm and microbial colonization with development of these diseases requires adequate removal of the bacterial factor from the dental implant surface. This is the reason why PDT was recommended, since its main objective is controlling disease progression through decontamination of infected surfaces [18, 19]. PDT is a simple and non-invasive technique that has proven to have antibacterial effects. Thus, this method can be recommended to treat pathological conditions involving bacterial etiology such as peri-implantitis and periodontal diseases. Although the expressive good results as an antimicrobial agent, further studies are needed to prove its efficiency in cases of PGCG around dental implants [7, 11, 20–22].

Consensus is reached in the literature that both clinical and radiographic follow-up is crucial. The recurrence rate of this lesion is not well determined yet; however, 4 out of 18 cases related showed recurrence of the PGCG [2, 3, 15]. In this context, a close follow-up is essential, monitoring irritant factors and encouraging an adequate hygiene from the patient, to identify as soon as possible any recurrence [23]. Our patient is still under clinical and radiographic monitoring.

In conclusion, reactive peri-implants exist. Thus, we need to make dental surgeons aware that dental implants need to be well planned and executed, with proper oral compliance, proper planning and execution, and periodic preventive maintenance, so if any of these lesions occurs, surgical excision with subperiosteal incision, scraping and adequacy of prosthetic components and crowns can be timely performed to avoid a dental implant failure.

## Data Availability

Not applicable. This is a case report without more data and materials.

## Abbreviations

PDT: Photodynamic therapy
PG: Pyogenic granuloma
PGCG: Peripheral giant cell granuloma

## References

[1] Atarbashi-Moghadam F, Atarbashi-Moghadam S, Namdari M, Shahrabi-Farahani S (2018). Reactive Oral lesions associated with a dental implants: a sistematic review. J Investig Clin Dent.

[2] Hirshberg A, Kozlovsky A, Schwartz-arad D, Mardinger O, Kaplan I (2003). Peripheral giant cell granuloma associated with dental implants. J Periodontal.

[3] Brown AM, Moraes PC, Sperandio M, Soares AB, Araújo VC, Passador-Santos F (2015). Peripheral Giant cell granuloma associated with a dental implant: a case report and review of the literature. Case Rep Dent.

[4] Bischof M, Nedir R, Lombardi T (2004). Peripheral giant cell granuloma associated with a dental implant. Int J Oral Maxillofac Implants.

[5] Tenore Gianluca, Mohsen Ahmed, Pompa Giorgio, Brauner Edoardo, Cassoni Andrea, Valentini Valentino, Polimeni Antonella, Romeo Umberto (2018). Gingival Reactive Lesions in Orally Rehabilitated Patients by Free Revascularized Flap. Case Reports in Dentistry.

[6] Cloutier Martin, Charles Makepeace, Carmichael Robert P., Sándor George K.B. (2007). An analysis of peripheral giant cell granuloma associated with dental implant treatment. Oral Surgery, Oral Medicine, Oral Pathology, Oral Radiology, and Endodontology.

[7] Ozden FO, Ozden B, Kurt M, Gündüz K, Günhan O (2009). Peripheral giant cell granuloma associated with dental implants: a rare case report. Int J Oral Maxillofac Implants.

[8] Hernandez Gonzalo, Lopez-Pintor Rosa M., Torres Jesús, de Vicente Juan Carlos (2009). Clinical Outcomes of Peri-Implant Peripheral Giant Cell Granuloma: A Report of Three Cases. Journal of Periodontology.

[9] Olmedo D.G., Paparella M.L., Brandizzi D., Cabrini R.L. (2010). Reactive lesions of peri-implant mucosa associated with titanium dental implants: a report of 2 cases. International Journal of Oral and Maxillofacial Surgery.

[10] Peñarrocha-Diago MA, Cervera-Ballester J, Maestre-Ferrín L, Peñarrocha-Oltra D (2012). Peripheral giant cell granuloma associated with dental implants: clinical case and literature review. J Oral Implantol.

[11] Pacifici A, Carbone D, Marini R, Sfasciotti GL (2015). Pacifici, L Clinical management of a peri-implant giant cell granuloma. Case Rep in Dentist.

[12] Galindo-Moreno P, Hernández-Cortes P, Rios R, Sanchez-Fernández E, Camara M, O’Valle F (2016). Immunophenotype of dental implant-associated peripheral giant cell reparative granuloma in a representative case report. J of Oral Implant.

[13] Scarano A, et al. Peripheral giant cell granuloma associated with dental implants. J Craniofac Surg. 2017;1. 10.1097/SCS.0000000000004281 29303864

[14] Cloutier M, Makepeace C, Carmichael RP, Sándor GKB (2017). An analysis of peripheral giant cell granuloma associated with dental implant treatment. Oral Surg Oral Med Oral Pathol Oral Radiol Endod.

[15] Scarano A, Lorusso C, Mortellaro C, Limongelli L, Tempesta A, Favia G (2018). Peripheral giant cell granuloma associated with dental implants. J Craniofac Surg.

[16] Hernandez G, Lopez-Pintor RM, Torres J, Vicente JC (2009). Clinical outcomes of peri-implant peripheral giant cell granuloma: a report of three cases. J Periodontol.

[17] Olmedo DG, Paparella ML, Brandizzi D, Cabrini RL (2010). Reactive lesions of peri-implant mucosa associated with titanium dental implants: a report of 2 cases. Int J Oral Maxillofac Surg.

[18] Bassetti M, Schär D, Wicki B, Erick S, Ramseier CS, Arweiler NB (2014). Anti-infective therapy of peri-implantitis with adjunctive local drug delivery or photodynamic therapy: 12-month outcomes of a randomized controlled clinical trial. Clin Oral Implants Res.

[19] Bombeccari GP, Guzzi G, Gualini F, Gualini S, Santoro F, Spadari F (2013). Photodynamic therapy to treat periimplantitis. Implant Dent.

[20] Ghanem A, Pasumarthy S, Ranna V, Kellesarian SV, Sbduljabbar T, Vohra F (2016). Is mechanical curettage with adjunct photodynamic therapy more effective in the treatment of peri-implantitis than mechanical curettage alone?. Photodiagn Photodyn Ther.

[21] Karimi MR, Hasani A, Khosroshahian S (2016). Efficacy of antimicrobial photodynamic therapy as an adjunctive to mechanical debridement in the treatment of peri-implant diseases: a randomized controlled clinical trial. J Lasers Med Sci.

[22] Manjunatha BS, Sutariva R, Nagamahita V, Dholia B (2014). Analysis of gingival biopsies in the gujarai population: a retrospective study. J Cancer Res Ther.

[23] Al Habashneh R, Asa'ad FA, Khader Y (2015). Photodynamic therapy in periodontal and peri-implant diseases. Quintessence Int.

